# Arrayed CdTeMicrodots and Their Enhanced Photodetectivity via Piezo-Phototronic Effect

**DOI:** 10.3390/nano9020178

**Published:** 2019-02-01

**Authors:** Dong Jin Lee, G. Mohan Kumar, P. Ilanchezhiyan, Fu Xiao, Sh.U. Yuldashev, Yong Deuk Woo, Deuk Young Kim, Tae Won Kang

**Affiliations:** 1 Quantum-Functional Semiconductor Research Center, Dongguk University-Seoul, Seoul 04623, Korea; jin514rin@naver.com (D.J.L.); dykim@dgu.edu (D.Y.K.); 2 Nano-Information Technology Academy (NITA), Dongguk University-Seoul, Seoul 04623, Korea; selvi1382@gmail.com (G.M.K.); xiaofu.04@foxmail.com (F.X.); shavkat@dongguk.edu (S.U.Y.); twkang@dongguk.edu (T.W.K.); 3 Department of Mechanical and Automotive Engineering, Woosuk University, Chonbuk 55338, Korea; wooyongd@woosuk.ac.kr

**Keywords:** CdTe microdots, Schottky barrier, photodetector, piezo-phototronic effect

## Abstract

In this paper, a photodetector based on arrayed CdTe microdots was fabricated on Bi coated transparent conducting indium tin oxide (ITO)/glass substrates. Current-voltage characteristics of these photodetectors revealed an ultrahigh sensitivity under stress (in the form of force through press) while compared to normal condition. The devices exhibited excellent photosensing properties with photoinduced current increasing from 20 to 76 μA cm^−2^ under stress. Furthermore, the photoresponsivity of the devices also increased under stress from 3.2 × 10^−4^ A/W to 5.5 × 10^−3^ A/W at a bias of 5 V. The observed characteristics are attributed to the piezopotential induced change in Schottky barrier height, which actually results from the piezo-phototronic effect. The obtained results also demonstrate the feasibility in realization of a facile and promising CdTe microdots-based photodetector via piezo-phototronic effect.

## 1. Introduction

Semiconductor nanostructures receive immense attention for their distinct physical properties and applications in high-performance nano devices, owing to their rationally designed surface and large surface to volume ratio [1,2,3]. Their unique morphological and crystalline characteristics make them very promising for designing and developing novel nanoscaled devices. In such cases, a control over their size and structure allowsus to tune their optical properties as well as their band gaps. Among many semiconductors, CdTe belonging to II–VI group is an optically active material with a band gap of 1.5 eV. This makes it a promising light-absorbing material for photovoltaics. Due to its excellent optical properties with high absorption coefficient and high specific power, CdTe is of great interest for application in optoelectronics devices such as photovoltaics, photodetectors, near-infrared detectors, gamma-ray detectors for medical imaging and lasers [4,5,6,7,8,9,10].

Due to its attractive bandgap and optical properties, CdTe in the nanostructures form is a potential candidate for the fabrication of a high-performance devices. Different morphologies of CdTe, such as nanoparticles, nanowires, nanorods, and nanotubes have been synthesized via a variety of methods including molecular beam epitaxy, chemical vapor deposition (CVD), physical vapor deposition, electrodeposition, closed spaced sublimation (CSS) method, and radio frequency(RF) magnetron sputtering [11,12,13,14,15,16,17,18,19,20]. CdTe solar cells on ultrathin glass substrates, yielding a high efficiency of 16.4%, have also been reported by Mahabaduge et al [21]. Recently, CdTe in the form of nanoribbons and nanowires were shown to exhibit significant photoresponse with high responsivity and gain [22,23]. 

In spite of the several advantageous optoelectronic properties of CdTe, their piezoelectric properties have seldomly been reported. Recently elastic and piezoelectric properties of zinc blende and wurtzite crystalline nanowire heterostructures were also reported [24,25,26]. Nonlinear piezoelectricity in CdTe was demonstrated by Corso et al by performing first principle calculation through density-functional theory (DFT) [27]. More recently, Hou et al. reported nanogenerator based on zinc blende CdTe micro/nanowires [28]. Inspired by these literatures, we showcase the piezopotential distribution on arrayed CdTe microdots under the application of external stress.

In this paper, we demonstrate the controlled growth of arrayed CdTe microdots using vapor phase epitaxy. The structural and morphology characterization are explained in detail. Additionally, investigations were also performed on the role of precursor to substrate distance over the controlled growth of CdTe microdots. Additionally, to showcase their potential for photoelectronic applications, we have investigated their electrical and photoelectronic properties by fabricating a photodetector using arrayed CdTe microdots. A strong photoelectric response was observed in the devices at room temperature without external power supply. The performance of this photodetector was significantly improved under the stress via piezo-phototronic effect. The photoresponsivity of the photodetector shows enhanced performance under stress than that of normal condition. The device displayed a detectivity of 1.68 × 10^11^ Jones under stress. This indicate that the performance of the arrayed CdTe microdots-based photodetector can effectively be enhanced through utilizing piezo-phototronic effect.

## 2. Materials and Methods

### 2.1. Synthesis of CdTe Films and Microdots

**Figure 1:**
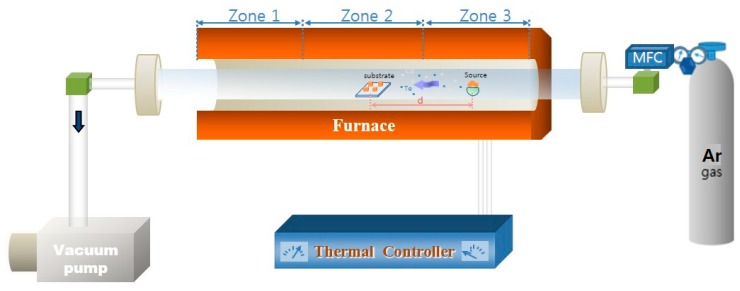
Apparatus for the deposition of the CdTe microdots arrays on Bi coated indium tin oxide (ITO)/glass.

Figure 1 shows the schematic experimental setup involved in the deposition of arrayed CdTe microdots on Bi coated indium tin oxide (ITO)/glass.CdTe films and microdots were selectively grown on Bi coated ITO/glass substrates by vapor phase epitaxy (VPE) method in a temperature controlled three-zone furnace. Bi films were first predeposited on precleanedITO/glass substrate via e-beam evaporator system as the specific substrate for selective growth of CdTe (20 nm). For this a grain type Bi metal source of 99.999% purity (Alfa Aesar product, Haverhill, MA, USA) was used. The film was deposited by using a shadow metal mask at a deposition rate of 1 Å/s at 2 × 10 ^−6^ Torr. The prepared substrate and CdTe bulk source (3.8 g, 99.888% purity Alfa Aesar product, Haverhill, MA, USA) was placed in Zone 2 (growth zone) and Zone 3 (source zone) of the VPE. The source to substrate distance was varied from 5 to 15 cm in order to obtain a clear micro dot pattern. The quartz chamber was pumped to a pressure of 2.4 × 10^−3^ Torr via rotary pump and unreacted (Ar) carrier gas was flown out at 60 sccm for 10 min before growth. The flow was maintained until the temperature had completely lowered after the growth. The temperatures of Zone 1 and Zone 2 were kept the same (250, 350 and 450 °C), while the temperature of Zone 3 was held at 600 °C. The temperature rise rate of all the samples was 10 degrees per minute and the growth time was 1 h. Bi was additionally coated on Al_2_O_3_ (111) and Si (100) substrates under similar growth conditions to determine their role as catalyst that effectively promotes the selectively growth of CdTe.

### 2.2. Device Fabrication

We also fabricated devices using CdTe microdots to observe the current changes due to photoreaction and physical forces. ITO/glass plates were placed on top of selectively grown CdTe microdots/Bi/ITO/glass samples (for physical contact and as the top electrode by fixing with a tape). The top and bottom electrode were connected to a wire and silver paste, respectively. The current was then measured according to the voltage when light irradiation and physical force were applied. We further studied the reproducibility of changes in current due to optical response and physical force. 

### 2.3. Characterization

The surface morphology/microstructure of CdTe samples were monitored through field-emission scanning electron microscopy (FESEM, Philips, Model: XL-30, Amsterdam, The Netherland). The microstructure of CdTe samples were inferred through X-ray diffraction (XRD, Bede scientific instruments, Model: Bede D1, Bowburn, UK) and Micro Raman spectrometer (DawoolAttonics, Model: Micro Raman System, Seongnam, Korea). Optical properties were obtained using a UV/VIS spectrophotometer (K LAB, Model: Optizen POP, Daejeon, Republic of Korea). A Keithley 617 semiconductor parameter analyzer (Tektronix, Model: Keithley 617, Beaverton, OR, USA) was employed to study the photoresponse of the device under solar simulator (Newport, AM1.5) (SERIC, Model: XIL-01B50KP, Tokyo, Japan). A specially designed spring-type soft stick was used to give a constant physical force to the device. The constant physical force was measured using a digital force gauge (Amittari, Model:FG-104, Guangdong, China) and the value was found to be 6.03 N/m^2^. A UV cut-off filter was employed in our experiments to exclude the influence of ITO on the photocurrent values of our devices. Hall effect measurements for CdTe microdots were provided in the Table S1.

## 3. Results and Discussion

**Figure 2:**
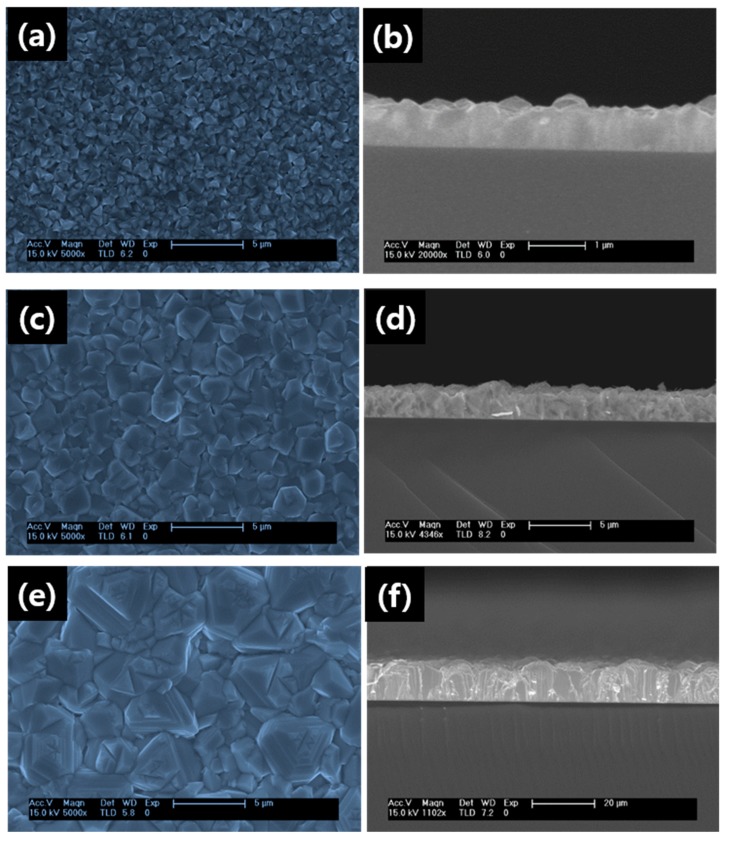
Scanning electron microscopy (SEM) images of CdTe grown at different substrate temperature (**a**) 250 °C (**c**) 350 °C (**e**) 450 °C along with their corresponding cross-sectional image (**b**,**d**,**f**).

Figure 2 shows the SEM and cross-sectional images of the CdTe films grown on Bi catalyst under different growth temperature. As seen from Figure 1, the surface topography of the film changes under different growth temperature. The films deposited at 250 °C (Figure 2a) shows uniform distribution of small grains on the substrate with many pinholes. On increasing the temperature to 350 °C, the grain size increases and fills the pin holes and result in continuous film (Figure 2c). The increase in the size of the grains might be related to the fact that the critical grain radius increases due to disappearance of the smaller grains and enhanced growth of larger grains. For further increase in the temperature (450 °C), the grain size increases substantially, and gets turned into elongated grain-like structure (Figure 2e). Compared with films deposited at temperature of 250 °C and 350 °C, the film uniformity has been improved for 450 °C.

To understand the effect of Bi film as seed layer for the formation of CdTe films, controlled experiments were conducted with and without Bi films on ITO substrates. The corresponding scanning electron microscopy (SEM) images of CdTe films grown under different growth temperatures are shown in Figure S1. In the early stages when the temperature was maintained at 250 °C nucleation of small grains of CdTe was observed on both with and without Bi film. When the temperature was increased to 450 °C, CdTe films was formed only on the Bi film. In this case no films were formed without Bi film, regardless of reaction condition. This indicates Bi film acts as a seed layer for the deposition of CdTe films. Additionally, different substrates such as Bi coated Al_2_O_3_ (111) and Si (100) substrate, were also employed to test the effect of substrate at the same growth temperature (Figure S2). No significant variation was observed in the results, as CdTe was formed only on the Bi coated area rather than uncoated area.

**Figure 3:**
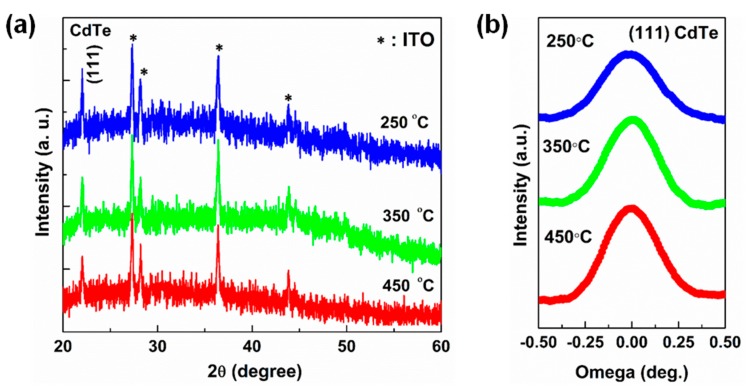
(**a**) XRD patterns of CdTe films grown under different substrate temperatures; (**b**) A rocking curve measurement for the 2θ = 23°diffraction peak.

Figure 3a displays XRD patterns of CdTe films grown under different temperatures. The diffraction peak observed at 23° corresponds to (111) plane of CdTe, suggesting that the crystal structure of CdTe films is zinc blende with a preferential orientation of the (111) plane, regardless of the growth temperature. The XRD pattern of the films deposited in the present study is consistent with that reported in the literature [29]. A rocking curve measurement for the 2θ = 23° diffraction peak was carried out for films grown under different temperatures (Figure 3b). The width of the rocking curve remains fairly narrow with a full width at half maximum (FWHM) of 0.356° for 250 °C and 0.321°, 0.310° for 350 °C and 450 °C (Figure 3b). The decrease in full width at half maximum (FWHM) with increasing temperature indicates that the crystallinity is improved. 

**Figure 4:**
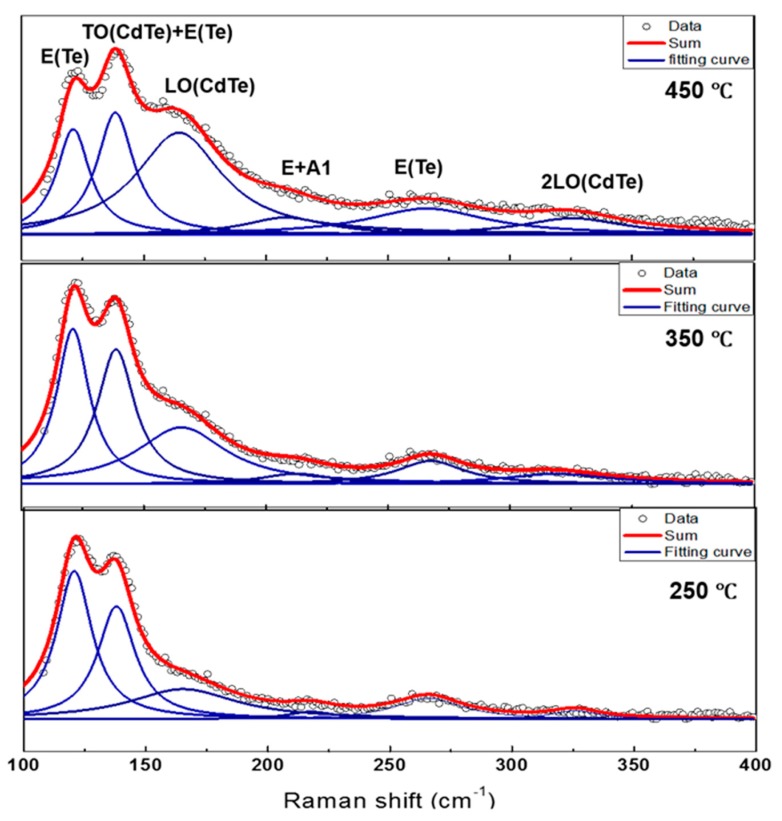
Raman spectrum (fitted using Lorentzian functions) of the CdTe films under different substrate temperature.

The Raman spectrum of CdTe films under different substrate temperature is shown in Figure 4. The spectrum fitted with Lorentzian functions exhibits six peaks at 120, 140, 160, 210, 260 and 330 cm^−1^. The vibration mode at 120 cm^−1^ and 260 cm^−1^ corresponds to elemental Tellurium phases [30]. The mode observed at 140 cm^−1^ could be attributed to combination of both transverse optical phonon (TO) and elemental Te [31]. The Raman modes at 162 and 330 cm^−1^ correspond to the longitudinal optical phonon (LO) and 2LO phonons of CdTe [32].Similarly, a broad band located at 210 cm^−1^ which we believe probably originates from the combination bands and we tentatively assign them to the overtones of E and A1 modes in Te [32]. Interestingly, by increasing the substrate temperature to 450 °C, (LO) and 2LO phonons modes of CdTe improved with reduction of Te related peaks and indicates improvement in crystallinity of CdTe.

**Figure 5:**
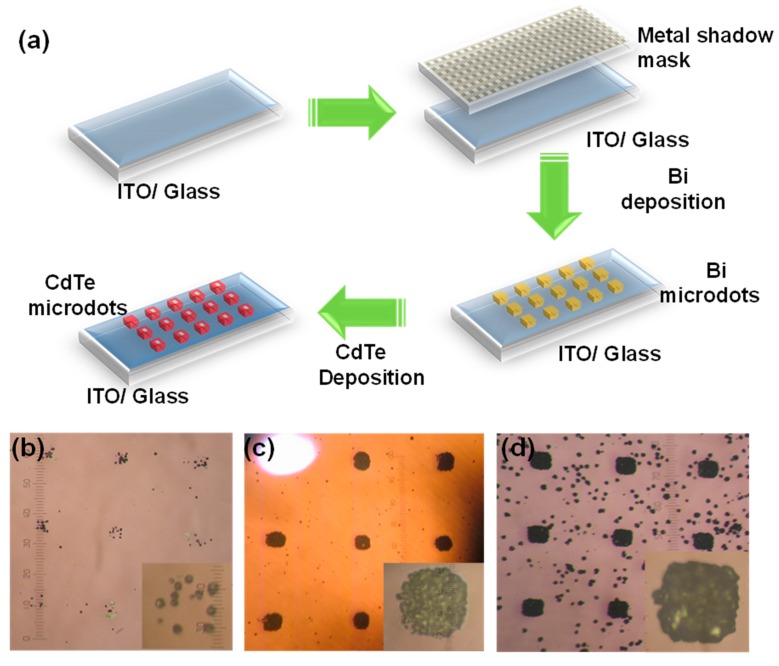
(**a**) Schematic of the fabrication process for the CdTe microdots arrays. Optical microscopy image of grown CdTe microdots arrays at different precursor to substrate distance (**b**) 15 cm, (**c**) 10 cm and (**d**) 5 cm.

The fabrication procedures for the CdTe microdots arrays photodetectors is illustrated in Figure 5a. Initially, 20 nm thick Bi film was deposited onto precleaned ITO glass substrate through a metal shadow mask using e-beam evaporation. The Bi film serves as seed layer for the growth of CdTe microdots-arrayed pattern. The CdTe microdots were grown using VPE. Figure 5b shows the optical microscopic image of CdTe microdots arrays. It has been shown that precursor to substrate distance (D) plays an important role in controlling the quality of CdTe films [33,34]. Interestingly the synthesized CdTe microdots were significantly different for substrate positions (D1 = 15 cm, D2 =10 cm, and D3 = 5 cm) as shown in Figure 5b–d. In case of sample (D1 = 15 cm), isolated and irregular CdTe nanoparticle were formed on the surface of Bi film (Figure 5b). When the distance (D1 = 15 cm) was kept longer from the CdTe source the concentration of the reactant species decreased, which resulted in slow reaction rate leading to uneven growth. Hence, the particle integration into films was incomplete. For sample (D2 = 10 cm), the film with high quality and smooth surface was formed on the Bi film (Figure 5c). In this case the concentration of the reactant species, gas flow, and reaction temperature weremore suitable for nucleation, thereby resulting inthe growth of high-quality film. For sample (D3 = 5 cm) the surface seems to be non-uniform with larger grain size (Figure 5d). Here, the distance (D3 = 5 cm) was close enough to the CdTe source, so a large number of disordered particles was formed due to rapid nucleation.Therefore, CdTe microdots grown at (D2 = 10 cm) possess smooth surface with higher quality than samples D1 and D3. Based on the above results, the precursor to substrate distances seems to have a huge influence on the formation of well-arrayed CdTe microdots.

**Figure 6:**
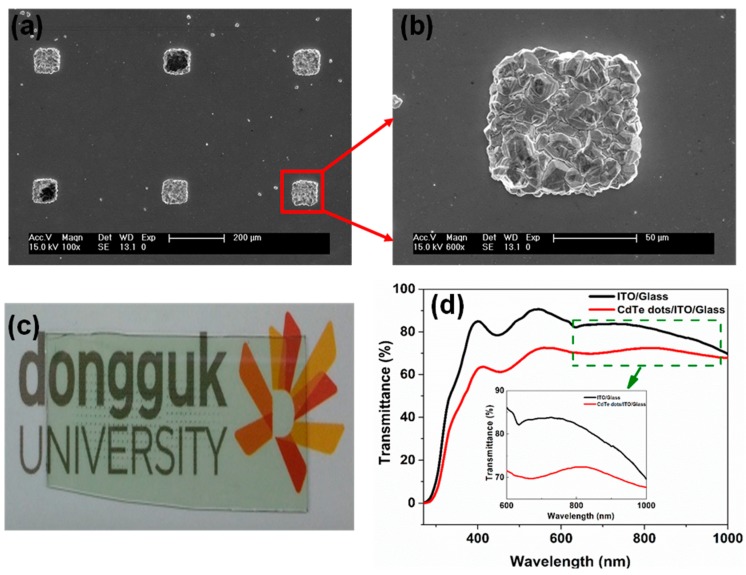
(**a**) SEM image of CdTe microdots arrays grown on Bi coated ITO substrate; (**b**) Enlarged version of the single microdots; (**c**) Photograph of the arrayed CdTe microdots films; (**d**) Transmittance spectra of CdTe microdots arrays film.

Figure 6a shows an SEM image of CdTe microdots arrays grown on Bi coated ITO substrate. As seen from Figure 6a, microdots were assembled in a perfect array and the distance between each dot was measured to be 470 μm (Figure S3). The enlarged version of the single microdots shows the size to be approximately 100 μm in diameter and has a smooth surface with high quality (Figure 6b). This result demonstrates that microdots could be arranged with precise control in size and position. Figure 6c shows the photograph of the arrayed CdTe microdots films. The corresponding transmittance spectra of arrayed CdTe microdots film are shown in Figure 6d. Here, the ITO glass shows about 80% transmittance across the visible range, while it drops to 72% when the CdTe micro dot film is deposited on ITO substrate. This decrease in transmittance is due to the absorption characteristics of the CdTe microdots film. The inset in Figure 6d shows the internal absorption around 825 nm in the case of CdTe microdots.

**Figure 7:**
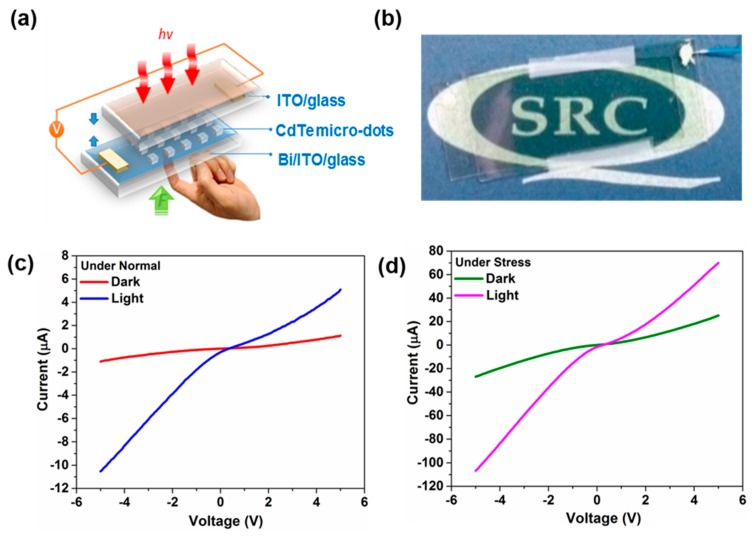
(**a**) Schematic of CdTe microdots array photodetectors; (**b**) Digital photographical image of a typical device; (**c**) Current-voltage (I–V) curve of the CdTe microdots array photodetectors under normal conditions in dark and illumination; (**d**) I–V characteristics of the device under stress (pressing condition) in dark and illumination.

Encouraged by the arrayed pattern and good crystallinity, we constructed a photodetector device based on CdTe microdots arrays film to explore its potential for optoelectronic applications. For the fabrication of photodetectors, we adopted a simple contact method using a sandwich structure of ITO glass that serves as transparent electrode and CdTe microdots on Bi-ITO as bottom electrode (See Experimental Section for the detailed fabrication process). Figure 7a shows schematic representation of CdTe microdots-arrayed photodetector on Bi/ITO substrates. For photoresponse measurement, light was illuminated to the device through the top ITO electrode (as shown in Figure 7a). A digital photographical image of a typical device is displayed in Figure 7b. Current-voltage (I–V) curves of the CdTe microdots-arrayed photodetector under normal conditions in dark and illumination are shown in Figure 7c. Here, the curves display asymmetric nonlinear behavior, suggesting the formation of a Schottky-like junction at ITO/CdTe microdots and CdTe/Bi/ITO interface. In other words, the device corresponds to two back-to-back Schottky junctions, with possibly slightly different Schottky barriers [35]. In contrast, the device exhibits strong photoresponse under illumination, indicating the contribution from photogenerated carriers. The dark current was measured to be 0.8 μA at a bias of 5 V. However, the current reaches to 5.3 μA at same bias voltage under illumination, indicating excellent sensitivity of the CdTe microdots. Under illumination, the absorption could mainly take place in CdTe, therefore most of the photogenerated electron-hole pairs are created at the CdTe/ITO junction. The photogenerated electrons-holes are quickly separated by the strong built-in electric field and will be collected at nearby electrodes. 

**Figure 8:**
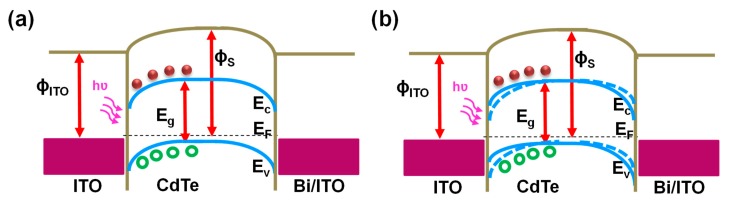
Schematic band diagrams of a CdTe microdots photodetector under (**a**) normal conditions and (**b**) with stress under illumination to illustrate the working mechanism of Piezo-phototronic effect-enhanced Photodetector performance.

To study the influence of piezoelectric effect on the performance of CdTe microdots array photodetectors, we applied an external stress (pressing the device from the back). We believe that, while pressing the sample, a stress could be completely applied to the CdTe microdots. The corresponding I–V characteristics of the device under such stress in dark and illumination is shown in Figure 7d. Here, the current flowing through the device increased compared to normal conditions. Such enhancement in currents under stress can be ascribed to the piezoelectric effects along CdTe microdots and ITO interface. The induced piezopotential at the CdTe microdots and ITO interface actually results in the change in the Schottky barrier height at CdTe/ITO interface and local electric field dominating the dark current. To confirm piezo-phototronic effect in CdTe microdots array photodetectors, I–V curves were measured by applying stress under illumination.Here, a notable enhancement in photocurrent (76 μA·cm^−2^) under illumination was observed while compared with that of dark current (20 μA·cm^−2^) under stress. It can be understood that the mechanically generated piezopotentials along the interface play a critical role in enhancing the performance of photodetector under illumination. When a stress is applied to the device, piezopotential is generated along the CdTe/ITO interface. The piezopotential at the CdTe/ITO interface, reduces the valence and conduction band energy level in CdTe. Meanwhile when the device is illuminated, the stronger electric field enhances the extraction and separation of photogenerated carriers, which results in enhanced photoresponse from the device (Figure 8b). The piezopotential induced under the stress condition plays a crucial role in increasing the performance of piezo-phototronic effect-based devices [36,37,38,39]. Similar experiments were also performed using CdTe thin films instead of CdTe microdots. However, we did not observe such a significant variation in the current values (Figure S4).

**Figure 9:**
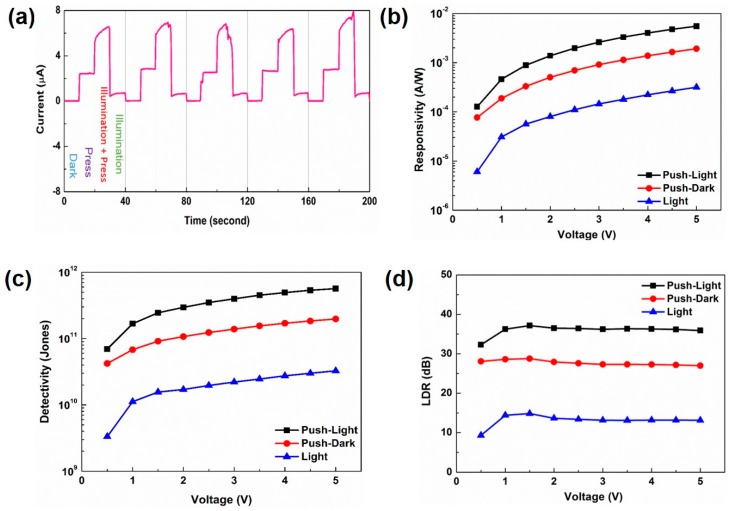
(**a**) Repeatable switching response of the device under 200 cycles with stress and illuminations. (**b**) Responsivity as a function of voltage under normal and stress condition. (**c**) Detectivity as a function of voltage under normal and stress condition. (**d**) Linear dynamic range LDR as a function of voltage under normal and stress condition.

Current–time (I–t) characteristics of the devices under normal and stress (pressing) conditions were recorded to investigate the piezo-phototronic effect on CdTe microdots devices (when external force wasapplied in a pulsed way to CdTe microdots device at 1 V). Figure S5 shows the current values of the device under normal and under stress condition. Here, in normal conditions the current values were found to be near zero and on applying stress to the device corresponding current of the CdTe device increased from 0.02 μA to 2.5 μA. It is worth mentioning that, the current always reached the same values at “press” state and recovered to the original current value when “no press” was applied to the substrate. As seen from the Figure 9a, under illumination on applying stress the photocurrent increases to 6.5 μA and then falls back to original current value. The time response of the device remains identical under normal and stress (pressing) conditions with no obvious degradation, indicating the excellent reproducibility and photocurrent stability of the CdTe microdots photodetector. 

Responsivity (R), is a critical parameter to determine the photoresponse performance of a photodetector. Photoresponsivity (R) is generally expressed based on the equation [40,41]

R = (I_p_ − I_d_)/P_light_ (1)

where I_p_ and I_d_ represent the photocurrent and dark current respectively,and P_light_ is incident light intensity. Figure 9b displays the responsivity of the photodetector under normal and stress upon illumination. It can be seen that the responsivity of the photodetector increases, with increasing bias voltage. The high photocurrent can be generated with the application of higher bias under illumination, since more charge carriers can pass through the Schottky junction, thereby resulting in enhanced responsivity. The value of photoresponsitivity was 3.2 × 10^−4^ A/W at (5 V). However, when a stress was applied to the device the photoresponsivity of the photodetector increases to one order on par with that of the normal condition. The value of photoresponsitivity increases to 5.5 × 10^−3^ A/W at (5 V). The significantly higher photoresponsivity values of CdTe microdots-arrayed photodetectors under stress condition is attributed to piezopolarization charges induced along interface between the CdTe and ITO. The induced piezoelectric effects under stress results in the effective modulation of the Schottky barrier height at CdTe/ITO interface which results in enhanced transport of photogenerated electrons and holes with reduced recombination probability for charge carriers. 

Detectivity (D*) is another important parameter to determine the performance of photodetector. The detectivity is expressed based on the following equation [42]

D* = RA^0.5 ^/(2eI_d_)^0.5^ (2)

where R, A, e and I_d_ represents the responsivity, effective area of the photodetector, electron charge, and dark current respectively. Figure 9c shows the D* of the CdTe microdots array photodetector under normal and stress condition. As seen from the Figure 9c, the detectivity (D*) was estimated to be 1.12 × 10^10^ Jones at (1V) for normal conditions. However, under pressing condition it increases to 1.68 × 10^11^ Jones, which reflects excellent sensitivity detection ability and outstanding performance in the photodetector. The higher values of detectivity observed under stress condition are attributed to the piezo-phototronic effect on the CdTe microdots.

Another critical parameter to determine the performance of photodetector is the linear dynamic range (LDR). The LDR is expressed by

LDR = 20·log(I_p_/I_d_) (3)

where I_p_ and I_d_ is the photocurrent and dark current, respectively. The LDR for normal and stress condition of the device under illumination is shown in Figure 9d. The calculated LDR for the CdTe microdots device under normal and stress conditions are 13 dB and 36 dB, respectively.The observed larger LDR value for stress conditions indicates that the CdTe microdots device opens up a great route to the next generation photodetectors.

## 4. Conclusions

In conclusion, we successfully fabricated controlled growth of CdTe microdots array photodetectors on Bi coated ITO substrates. The significant enhancement in the electrical transport and photosensing properties of CdTe microdots array under stress condition can be ascribed to the piezo potentials induced along the CdTe/ITO interface. The induced piezopotential results in a stronger local electric field, resulting in enhanced separation and extraction of the photoexcited carriers at the CdTe/ITO interface. This results in the enhancement of photoresponse of the Schottky junction. Under the application of stress, the photoresponsivities of the CdTe microdots array photodetectors are increased from 3.2 × 10^−4^ A/W to 5.5 × 10^−3^ A/W, respectively, compared to that of normal condition. These results demonstrate that the photoresponse performance of CdTe microdots array photodetectors can be effectively enhanced using piezo-phototronic effect and provide a feasible approach to extend new design concepts using CdTe with different morphologies and broaden the scope of its potential in optoelectronics applications.

## Supplementary Materials

The following are available online at https://www.mdpi.com/2079-4991/9/2/178/s1, Figure S1. SEM images of CdTe grown with and without Bi films under different substrate temperature (a) 250 °C; (b) 350 °C and (c) 450 °C, Figure S2. Photograph images of CdTe grown with and without Bi films on (a) Al2O3 (b) Si and (c) ITO substrate, Figure S3. SEM image of CdTe microdots arrays grown on Bi coated ITO substrate, Table S1: Hall effect measurements for CdTe microdots, Figure S4. Current vs. time characteristics of CdTe thin films under with press and without press conditions, Figure S5. Current values of the device under normal and under stress (pressing) condition.
