# Supplementary material for: Arrayed CdTeMicrodots and Their Enhanced Photodetectivity via Piezo-Phototronic Effect
Source: Nanomaterials (Basel). 2019 Feb 1;9(2):178. doi: 10.3390/nano9020178 (PMC6409905; doi:10.3390/nano9020178)
Supplement: Supplementary file 1 [file nanomaterials-09-00178-s001.pdf]

Supplementary Materials

Arrayed CdTeMicrodots and Their Enhanced Photodetectivity via Piezo-Phototronic Effect

Dong Jin Lee [1], G. Mohan Kumar [2], P. Ilanchezhiyan[2,*], Fu Xiao [2], Sh.U. Yuldashev[2], Yong Deuk Woo [3], Deuk Young Kim [1] and Tae Won Kang [2]

1 Quantum-Functional Semiconductor Research Center, Dongguk University-Seoul, Seoul 04623, Korea; jin514rin@naver.com (D.J.L.); dykim@dgu.edu (D.Y.K.)
2 Nano-Information Technology Academy (NITA), Dongguk University-Seoul, Seoul 04623, Korea; selvi1382@gmail.com (G.M.K.); xiaofu.04@foxmail.com (F.X.); shavkat@dongguk.edu (S.U.Y.); twkang@dongguk.edu (T.W.K.)
3 Department of Mechanical and Automotive Engineering, Woosuk University, Chonbuk 55338, Korea; wooyongd@woosuk.ac.kr (Y.D.W.)
* Correspondence: ilancheziyan@dongguk.edu

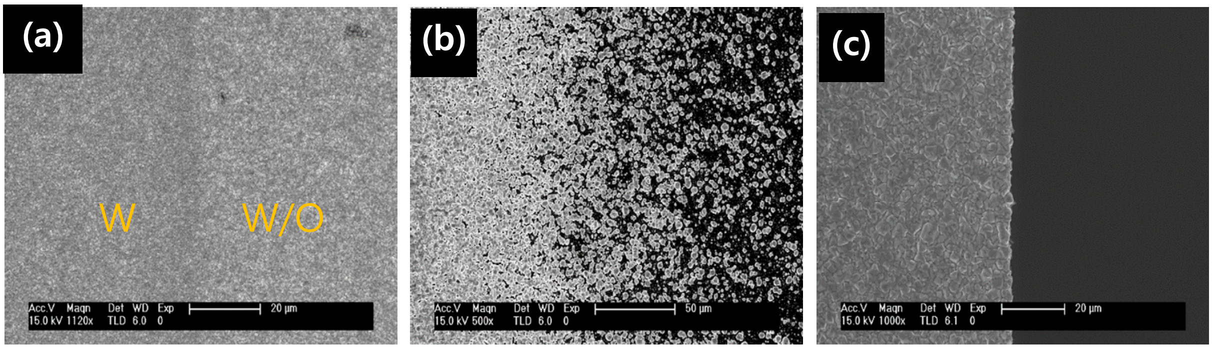

Figure S1. SEM images of CdTe grown with and without Bi films under different substrate temperature (**a**) 250 °C; (**b**) 350 °C and (**c**) 450 °C.

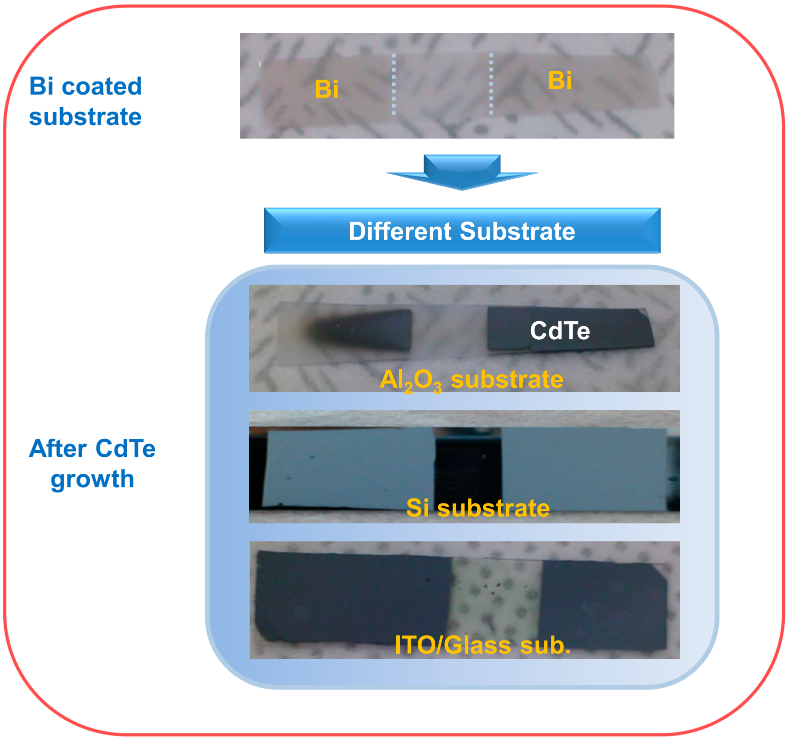

Figure S2. Photograph images of CdTe grown with and without Bi films on (**a**) Al_2O_3 (**b**) Si and (**c**) ITO substrate.

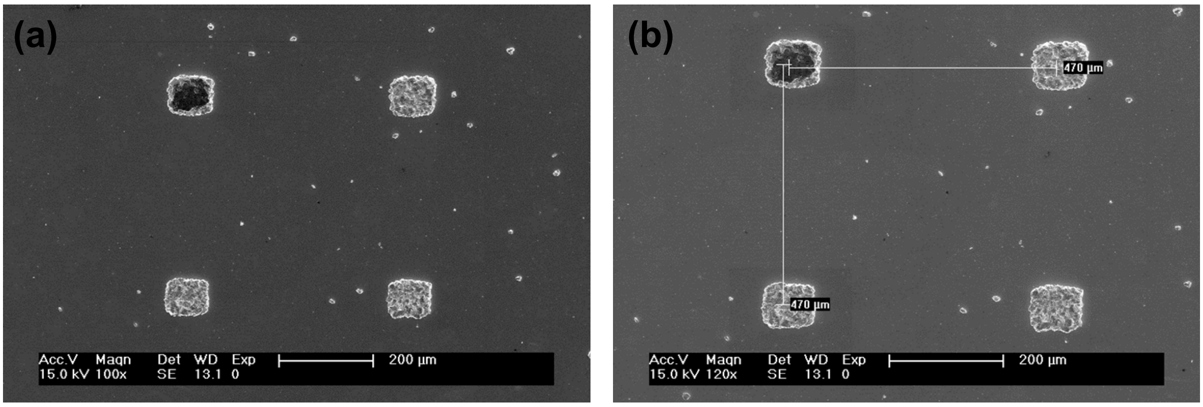

Figure S3. SEM image of CdTe microdots arrays grown on Bi coated ITO substrate.

Table S1: Hall effect measurements for CdTe microdots.

	R_H (m^2/C)	μ_H (cm^2/V.s)	σ (Ω.cm)	n_H (cm^{-3})	Type
CdTe (450°C)	27.3	17.86	3.48×10^4	2.28×10^{14}	p-type

All data including Hall coefficient (R_H), conductivity (σ), carrier mobility (μ_H), and carrier concentration (n_H) for the CdTe microdots are shown in the Table I.

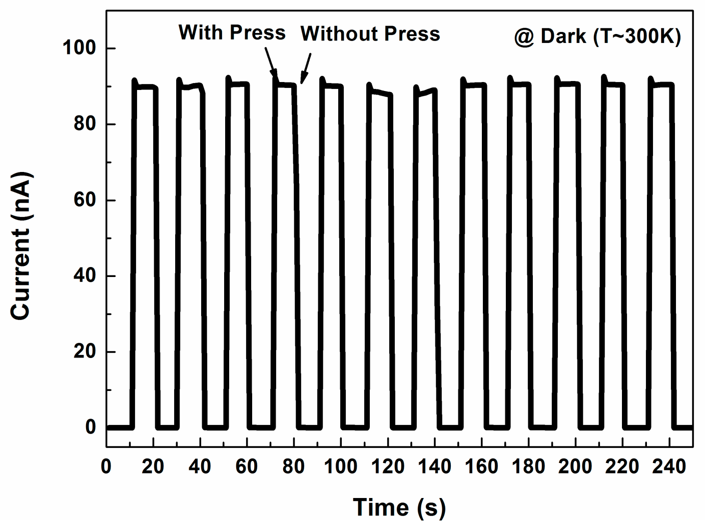

Figure S4. Current vs. time characteristics of CdTe thin films under with press and without press conditions.

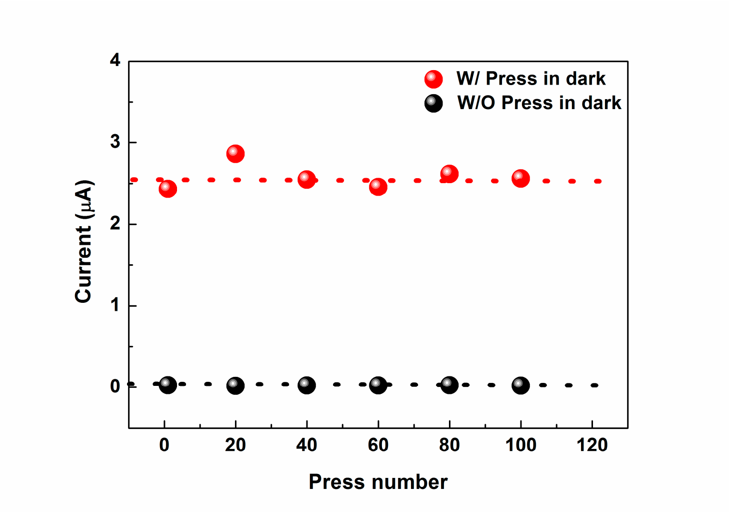

Figure S5. Current values of the device under normal and under stress (pressing) condition
